# Chaos Analysis of Urban Low-Carbon Traffic Based on Game Theory

**DOI:** 10.3390/ijerph18052285

**Published:** 2021-02-25

**Authors:** Xiaohui Wu, Ren He, Meiling He

**Affiliations:** School of Automotive and Traffic Engineering, Jiangsu University, Zhenjiang 212013, China; heren@mail.ujs.edu.cn (R.H.); hemeiling@ujs.edu.cn (M.H.)

**Keywords:** low-carbon traffic, game theory, chaos analysis, carbon emissions

## Abstract

Developing urban low-carbon traffic is an effective measure to reduce traffic carbon emissions, which are important parts of greenhouse gas. In order to understand the development characteristics and regular patterns of urban low-carbon traffic, we present a game model that enables us to predict the possible range of travel mode choice and the impact of low-carbon awareness. Through chaos analysis and simulation of the model, the authors come to realize that the proportions of travel mode choice can reach an equilibrium under a certain urban traffic system. This equilibrium is related to low-carbon awareness and the situation of the urban traffic system. The research we have done suggests that in small cities with undeveloped traffic systems, the most effective measure to achieve urban low-carbon traffic is to increase the comprehensive costs of high-carbon travel. However, in big cities with developed traffic systems, raising low-carbon awareness of residents can greatly increase the proportion of low-carbon travelers and improve the stability of travel mode choice. The results could provide development strategies and policy suggestions for urban low-carbon traffic and reduce the adverse impact of urban traffic emissions on public health.

## 1. Introduction

Generally speaking, urban transportation is one of the main sources of traffic carbon emissions [1,2]. The greenhouse effect and sustainable development issues caused by carbon emissions have received global attention. Urban low-carbon transport has been a hot topic in many studies. The main research directions are to study the impact of low-carbon transport system design [3,4,5] and policy guidance [6,7,8] on the traffic carbon emissions of various types of cities. In fact, both transport system design and policy guidance are reflections of the travel mode choices. Therefore, it is necessary to research the travel mode choices and development trends of urban travelers in the context of low -carbon, which is one of the key factors to reduce traffic carbon emissions.

Since the 1994 Nobel Prize in Economics was awarded to three game theory experts, more and more researchers have begun to use game theory to study traffic problems [9,10]. Game theory considers the predicted and actual behaviors of individuals in the game and studies their optimization strategies. It is used in traditional traffic revenue distribution [11,12,13], and has made achievements in emerging research areas such as internet of vehicles control strategies [14], path optimization [15], and system evaluation [16]. Some studies have also accomplished certain achievements by using game theory in the field of low-carbon transportation [17]. By studying the three-party game of government, company, and consumer, the development path and trend of electric vehicles are demonstrated [18]. There are also some pollution path games for freight carbon emissions through analysis of distance and network flow [19]. On the other hand, chaos theory that is a method of qualitative thinking and quantitative analysis is suitable for analyzing the influencing factors and predicting the direction of traffic flow. Chaos theory shows great superiority in eliminating the interference factors of traffic flow prediction [20] and improving the prediction accuracy [21,22].

Therefore, game theory combined with chaos analysis have certain advantages in the study of travel mode and routes choices, as well as the changing trend of these choices. However, at present the established structural equation model (SEM) based on the theory of planned behavior (TPB) and the value–belief–norm theory (VBN) are the main methods to analyze and predict travel mode choices [23,24,25]. However, in order to guide the sustainable development of urban transport, some researchers have proposed to use evolutionary game theory as a framework to study the travel mode choices and the impacts of transportation policy changes on the equilibrium of traffic systems [26]. Some advanced research methods, such as geographic information system (GIS), are also used to improve the accuracy of choices prediction [27]. In comparison, most of these studies do not focus on low-carbon traffic and SEM is still the main method in the study of travel mode choices considering low-carbon factors. The advantages of game theory in considering these problems have not been fully exploited. Furthermore, the chaos analyses of the game equilibrium stable point are mostly seen in some economic studies [28]. These studies generally use the game theory model of outputs or prices competition to analyze a nonlinear dynamic system with different strategies. The existence and stability of the output equilibrium point of two competitive products are the focus of discussion. This is similar to the problem that travelers choose the mode of transportation according to their own comprehensive judgment. The approaches that could effectively analyze the influence of uncertain factors in complex systems provide new references for urban low-carbon travel studies.

Overall, previous studies mainly focused on the prediction of the proportion of travel modes, but the research on the conditions to achieve and maintain this proportion was insufficient. Therefore, based on the comprehensive traffic conditions of cities, the main purpose of this study is to use game theory and chaos theory to establish a model for travel mode choices and to explore the stable equilibrium solution and chaos characteristics of these choices. Low-carbon and high-carbon travelers who play games according to certain strategies choose their travel mode in order to maximize the comprehensive travel payoffs. The development trend and stability of these choices under different complex conditions are exactly what we are most concerned with. This research is conducive to a better understanding of the game behavior in low-carbon traffic from the macro perspective, which can predict the development trend of travel mode choices under different conditions, provide development strategies and policy suggestions for urban low-carbon traffic, reduce long-term traffic carbon emissions, and promote the improvement of urban air quality.

## 2. Materials and Methods

### 2.1. Low-Carbon Travel Awareness Survey

In order to describe low-carbon travel awareness (ω), we issued an online questionnaire survey on a professional platform, named ‘Wenjuanxing’, in China. The importance of the low-carbon travel factor compared with traditional travel factors is divided into 0 to 10 levels. Level 0 (Lv 0), which is ω=0, means that low-carbon factor is not considered at all and has no impact on travel behavior. Level 10 (Lv 10), which is ω=1, means that the low carbon factor is as important as the sum of traditional travel factors. From the perspective of low-carbon travel choice, this is another extreme situation contrary to completely ignoring low-carbon factors. After the investigation, we collected 1011 valid questionnaires in July 2020. In order to be statistically significant, we listed cities with more than 100 questionnaires, as shown in Table 1.

**Figure 1:**
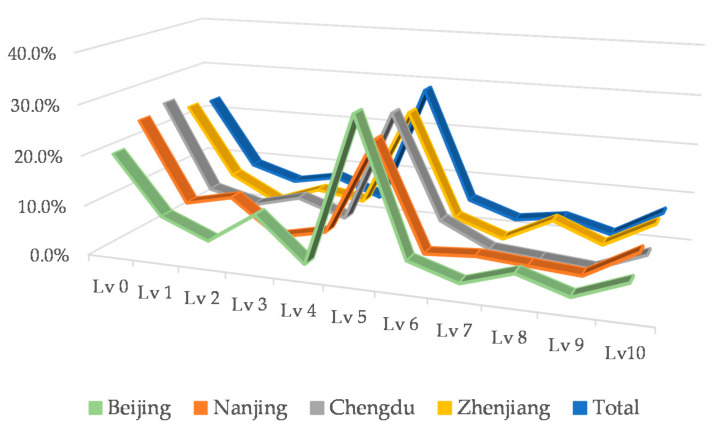
Comparison of low-carbon awareness percentage of urban travelers.

To increase comparability, we converted the four cities and total data into percentages, as shown in Figure 1.

By comparing the data in Figure 1, it is found that the curve of the total data is basically in the median, which can reflect the general situation of travelers’ low-carbon awareness. Therefore, this group of data is selected as the middle awareness (M-A) of low-carbon travel. We also noticed that Chengdu has the highest proportion of travelers who do not consider low-carbon factor at all, and the lowest proportion of those who think low-carbon factor is the most important. Therefore, Chengdu’s data could be selected as the low awareness (L-A) of low-carbon travel. In addition, regardless of the overall low-carbon travel awareness of a city, the proportion of Lv 5 is dominant. Based on these characteristics and data, we also set the typical probability distribution of high awareness (H-A) of low-carbon travel, as shown in Table 2.

### 2.2. Game Theory Model

The Duopoly Stackelberg model describes that in a monopoly industry, two different companies which produce the same products can obtain the maximum profit by adjusting production strategy. From the three most important elements of game theory, players, strategies, and payoffs, we find that the process of urban low-carbon travel is very consistent with the process described by the model. Firstly, low-carbon travelers and high-carbon travelers are the monopoly players of urban travel. Although the characteristics are different, their essence is to carry out transport activities and accept transport services, which basically corresponds to the similar products. Secondly, they implement strategies similar to output adjustments by choosing different travel modes. The macro performance of these choices is the quantity of travelers. Thirdly, According to the generalized Wordrop principle, the game process of travelers estimating their comprehensive payoff and making strategic choices is also the process of approaching the optimal payoff of the system [29]. This is consistent with the objective of achieving the optimal payoffs of the companies. For these reasons, we consider that the Duopoly Stackelberg model could depict the traffic mode appropriately, and it is a valuable exploration to study the regular pattern of low-carbon travel based on this model.

The travel modes of urban travelers are mainly divided into low-carbon modes (such as metro and bus) and high-carbon modes (such as private car and taxi). Taking low-carbon travel awareness as an entry point, we established an improved Duopoly Stackelberg model of low-carbon and high-carbon travelers under certain traffic conditions and analyzed the stability of equilibrium points through chaos analysis. According to the theory of transportation economics, the relationship between quantity and comprehensive payoffs of travelers could be expressed by the reverse demand curve [30], which is often used by transport [31] and game theory research [32,33].

(1) $$p_{i}=a-b_{i}q_{i}-dq_{j,}\;\;\;i,j=1,2\;\;\;i\neq j$$

The inverse demand curve expressed in Equation (1) establishes a functional relationship that $p_{i}$ (comprehensive payoffs of travelers) decreases with the increase of $q_{i,j}$ (scale of travelers’ quantity). The comprehensive payoffs of travelers refer to all transport utility service as a product. $a,\;b_{i}$ and d are demand curve parameters, which form a bounded closed convex set in the first quadrant for finding the equilibrium solution of the game. a limits the maximum value of the ordinate of this set and the combination of a with $b_{i}$ and d also limits the value of abscissa. $b_{i}$ and d indicate the influence coefficient of travelers’ quantity in the same and different travel mode on the comprehensive payoffs respectively.

The adjusted comprehensive travel cost ${\tilde{c}}_{i}$ can be expressed by Equations (2) and (3).

(2) $${\tilde{c}}_{i}=(1+\omega_{i})c_{i}$$

(3) $$\omega_{i}={\left(-1\right)}^{i}\mu_{\omega }$$

$c_{i}$ represents the traditional comprehensive travel cost, such as travel time, price, and comfort [34]. There is an evident difference in $c_{i}$ between low-carbon and high-carbon travel. ω symbolizes the probability and degree of the impact of low-carbon awareness on the comprehensive travel cost. The probability distribution of low-carbon awareness ω is shown in Table 2. Through statistical analysis, in L-A, mathematical expectation of ω is $\mu_{\omega }=0.313$; in M-A, $\mu_{\omega }=0.376$; in H-A, $\mu_{\omega }=0.480$. We use $\mu_{\omega }$ to represent the average low-carbon awareness of the society in the corresponding situation. When urban residents travel in low-carbon mode, let *i* = 1. Considering the impact of low-carbon awareness, it can be considered that their comprehensive travel cost in the travel game is adjusted to ${\tilde{c}}_{i}$, with an average reduction of $c_{i}\mu_{\omega }$. When urban residents travel in high-carbon mode and *i* = 2, it would have a corresponding increase.

The net payoffs (${\tilde{p}}_{i}$) and marginal payoffs of travelers can be expressed as:

(4) $${\tilde{p}}_{i}=(a-b_{i}q_{i}-dq_{j})q_{i}-{\tilde{c}}_{i}q_{i}$$

(5) $$\frac{\partial {\tilde{p}}_{i}}{\partial {\tilde{q}}_{i}}=a-2b_{i}q_{i}-dq_{j}-{\tilde{c}}_{i}$$

Assume that Equation (5) = 0, the optimal quantity of travelers can be expressed as $q_{i}$:

(6) $$q_{i}=\frac{a-dq_{j}-{\tilde{c}}_{i}}{2b_{i}}$$

High-carbon travelers generally have advantages in some aspects, such as income and car ownership, and they have higher initiative in the choice of travel mode [35]. Therefore, the low-carbon traffic problem is formulated as a Stackelberg game where low-carbon travelers are the leaders and low-carbon travelers are followers [36]. In this situation, the strategy of low-carbon travelers can be regarded as the process of pursuing the maximum net payoffs based on observing the quantity of high-carbon travelers. Therefore, the adjustment strategy of low-carbon travelers could be expressed as:

(7) $$q_{1}(t+1)=\frac{a-dq_{2}(t)-{\tilde{c}}_{1}}{2b_{1}}$$

If the travel payoffs cannot satisfy the expectations of high-carbon travelers, they might take the initiative to change their strategy. We consider that high-carbon travelers adjust their strategies with a certain rate *k* through the estimation of marginal payoffs [33]. Therefore, the adjustment strategy of high-carbon travelers could be expressed as:

(8) $$q_{2}(t+1)=q_{2}(t)+kq_{2}(t)[a-2b_{2}q_{2}(t)-dq_{1}(t)-{\tilde{c}}_{2}]$$

The discrete dynamic system of urban residents’ low-carbon travel game can be expressed as:

(9) $$\left\{\begin{array}{l} q_{1}(t+1)=\frac{a-dq_{2}(t)-{\tilde{c}}_{1}}{2b_{1}}\\ q_{2}(t+1)=q_{2}(t)+kq_{2}(t)[a-2b_{2}q_{2}(t)-dq_{1}(t)-{\tilde{c}}_{2}] \end{array}\right.$$

### 2.3. Stability Analysis of Game Theory Model

The Jacobian determinant of Equation (9) is:

(10) $$\left[\begin{array}{cc} 0 & \frac{-d}{2b_{1}}\\ -kdq_{2} & 1+k(a-4b_{2}q_{2}-dq_{1}-{\tilde{c}}_{2}) \end{array}\right]$$

Assume that $q_{i}(t)=q_{i}(t+1)$, the equilibrium point $E_{1}$, $E_{2}$ of Equation (10) can be expressed as:

(11) $$E_{1}=(q_{1}^{*},0),\;\;\;E_{2}=(q_{1}^{**},q_{2}^{**})$$

where

(12) $$\begin{array}{l} q_{1}^{*}=\frac{a-{\tilde{c}}_{1}}{2b_{1}}\\ q_{1}^{**}=\frac{a(2b_{2}-d)+d{\tilde{c}}_{2}-2b_{2}{\tilde{c}}_{1}}{4b_{1}b_{2}-d^{2}}\\ q_{2}^{**}=\frac{a(2b_{1}-d)+d{\tilde{c}}_{1}-2b_{1}{\tilde{c}}_{2}}{4b_{1}b_{2}-d^{2}} \end{array}$$

So, the Jacobian matrix of the equilibrium point $E_{1}$ can be expressed as:

(13) $$J(E_{1})=\left[\begin{array}{cc} 0 & \frac{-d}{2b_{1}}\\ 0 & 1+k(a-dq_{1}^{*}-{\tilde{c}}_{2}) \end{array}\right]$$

The eigenvalues of Equation (13) are $\lambda_{1}=0$, $\lambda_{2}>1$, so $E_{1}$ is an unstable saddle point.

The Jacobian matrix of the equilibrium point $E_{2}$ can be expressed as:

(14) $$J(E_{2})=\left[\begin{array}{cc} 0 & \frac{-d}{2b_{1}}\\ -kdq_{2}^{**} & 1+k(a-4b_{2}q_{2}^{**}-dq_{1}^{**}-{\tilde{c}}_{2}) \end{array}\right]$$

The characteristic equation of matrix $J(E_{2})$ is:

(15) $$\lambda^{2}-Tr(J)+Det(J)=0$$

From Equations (14) and (15), the following equation can be derived:

(16) $$Tr(J)=1+k(a-4b_{2}q_{2}^{**}-dq_{1}^{**}-{\tilde{c}}_{2})$$

(17) $$Det(J)=-\frac{kd_{2}q_{2}^{**}}{2b_{1}}$$

Obviously, ${\left(Tr(J)\right)}^{2}-4Det(J)>0$. In order to make $E_{2}$ locally stable, it is necessary to satisfy the following conditions:

(18) $$\left\{\begin{array}{l} 1+Tr(J)+Det(J)>0\\ 1-Tr(J)+Det(J)>0\\ 1-Det(J)>0 \end{array}\right.$$

From Equation (17), 1−Det(J)>0 can be easily derived.

According to the above, it can be seen that $q_{1}^{**}>0,\;q_{2}^{**}>0$, $d\in (0,b_{i})$. Through derivation, the corresponding equation goes here:

(19) $$1-Tr(J)+Det(J)=k(a-dq_{1}^{*}-{\tilde{c}}_{2})(1-\frac{d^{2}}{4b_{1}b_{2}})>0$$

Therefore, the local stability condition of $E_{2}=(q_{1}^{**},q_{2}^{**})$ is:

(20) 1+Tr(J)+Det(J)>0

Putting Equations (16) and (17) into Equation (20), the corresponding equation goes here:

(21) $$4b_{1}(4b_{1}b_{2}-d^{2})-k(4b_{1}b_{2}+d^{2})[a(2b_{1}-d)+d{\tilde{c}}_{1}-2b_{1}{\tilde{c}}_{2}]>0$$

Therefore, Equation (21) represents the conditions for the existence of locally stable points in the game system.

## 3. Results

According to Equation (21), the stable region of Nash equilibrium point $E_{2}$ can be expressed by k∈[0,τ], where

(22) $$\tau =\frac{4b_{1}(4b_{1}b_{2}-d^{2})}{\left(4b_{1}b_{2}+d^{2})[a(2b_{1}-d)+d{\tilde{c}}_{1}-2b_{1}{\tilde{c}}_{2}\right]}$$

Then, we calibrate the above parameters based on the analysis of urban traffic characteristics, and use MATLAB for numerical simulation.

### 3.1. Parameter Calibration

Because the parameters of the game model are dimensionless, the calibration of the parameters is very important. We analyze the relative values of the model parameters in different types of cities, and then refer to the literatures to calibrate the parameters.

Cities with developed traffic systems are generally larger and more populous than cities with undeveloped traffic systems. Therefore, a, related to the boundary of game closed convex set, is larger in cities with developed traffic systems. Comprehensive payoffs decrease with the increase of the quantity of travelers, and the increase in the same travel mode has a greater impact than the increase in the different travel mode, so $b_{i}>d$. Meanwhile, due to the large transport capacity of metros, the quantity increase of low-carbon travel in big cities has less impact on the comprehensive payoffs. Many studies have also discussed the traditional comprehensive travel cost $c_{i}$ in different situations [34]. The traditional comprehensive cost of low-carbon travel in cities with developed traffic systems is lower than that in other cities, while the cost of high-carbon travel is higher than that in others [37].

Based on the above analysis and relative studies [26,33], the parameters of cities with developed traffic systems are calibrated as $a=30,\;d=0.3,\;c_{1}=2,\;c_{2}=7,\;b_{1}=0.8,\;b_{2}=0.6$ and the parameters of cities with undeveloped traffic systems are calibrated as $a=15,\;d=0.5,\;c_{1}=3,\;c_{2}=5,\;b_{1}=0.7,\;b_{2}=0.6$.

### 3.2. Simulation Results

#### 3.2.1. Simulation Results of Cities with Developed Traffic Systems

According to the parameter calibration of cities with developed traffic systems, the results of travelers’ selection under L-A and H-A of low-carbon travel awareness should be simulated.

**Figure 2:**
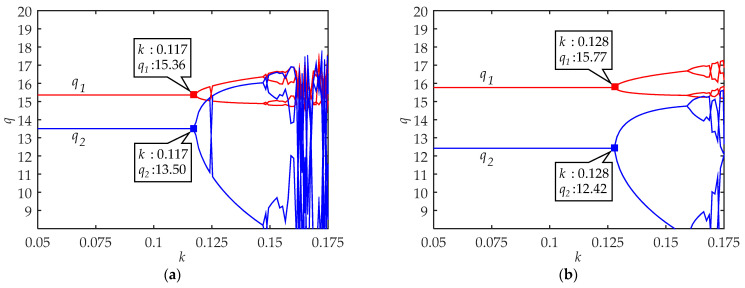
The system equilibrium quantities of low-carbon travel ($q_{1}$) and high-carbon travel ($q_{2}$) in cities with developed traffic systems: (**a**) Under L-A of low-carbon travel; (**b**) under H-A of low-carbon travel.

**Figure 3:**
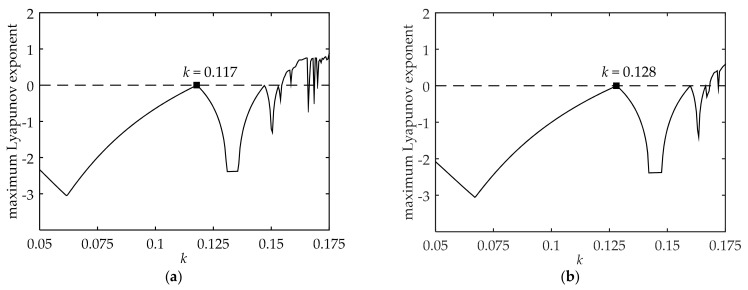
Maximum Lyapunov exponent of the system in cities with developed traffic systems: (**a**) Under L-A of low-carbon travel; (**b**) inder H-A of low-carbon travel.

As shown in Figure 2a and Figure 3a, $q_{1}$ and $q_{2}$ in cities with developed traffic systems under L-A of low-carbon travel could maintain certain equilibriums before *k* < 0.117. When *k* > 0.117, by comparing with the maximum Lyapunov exponent curve, we find that the traveler’s choice system falls into chaos and the stable equilibrium solution cannot be obtained. Meanwhile, Figure 2b and Figure 3b show that the critical value of *k* is equal to 0.128 under L-A of low-carbon travel. On the other hand, Figure 2a and Figure 2b show that with the growth of low-carbon awareness, $q_{1}$ also increases while $q_{2}$ decreases. The decrease of $q_{1}$ is slightly more than the increase of $q_{2}$.

#### 3.2.2. Simulation Results of Cities with Undeveloped Traffic Systems

**Figure 4:**
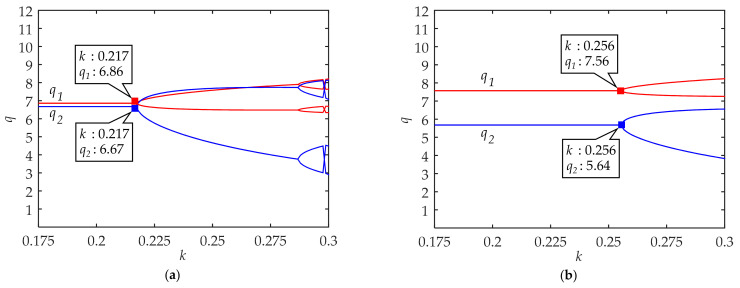
The system equilibrium quantities of low-carbon travel ($q_{1}$) and high-carbon travel ($q_{2}$) in cities with undeveloped traffic systems: (**a**) Under L-A of low-carbon travel; (**b**) under H-A of low-carbon travel.

The results of travelers’ selection under L-A and H-A of low-carbon travel awareness in cities with undeveloped traffic systems are as follow:

As shown in Figure 4, $q_{1}$ and $q_{2}$ in cities with undeveloped traffic systems under L-A of low-carbon travel could maintain certain equilibriums before *k* < 0.217. Similar to cities with developed traffic systems, when *k* > 0.217, the system will fall into chaos and the critical value of *k* increases in Figure 4b. Comparing Figure 4a and Figure 4b, we also find that with the growth of low-carbon awareness, $q_{1}$ increases while $q_{2}$ decreases.

An evident change between Figure 4 and Figure 2 is that the critical value of *k* increases. It means that the system is more stable, and the cities with undeveloped traffic systems have stronger adaptability to the change of traffic conditions. Therefore, in order to explore other regular patterns of low-carbon travel, we simulate the impacts of $c_{1}$ and $c_{2}$ changes on the system in these cities with medium low-carbon awareness.

#### 3.2.3. Simulation of the Impact of $c_{1}$ and $c_{2}$ in Cities with Undeveloped Traffic Systems

**Figure 5:**
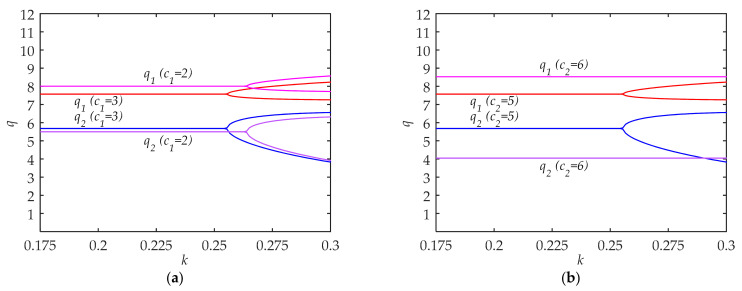
The system equilibrium quantities of low-carbon travel ($q_{1}$) and high-carbon travel ($q_{2}$) in cities with undeveloped traffic systems under M-A of low-carbon travel: (**a**) $c_{1}$ decreases from 2 to 3, and other parameters are fixed; (**b**) $c_{2}$ increases from 5 to 6, and other parameters are fixed.

As shown in Figure 5, changing the value of $c_{1}$ from 3 to 2 and $c_{2}$ from 5 to 6 both increase $q_{1}$ and decrease $q_{2}$. The increase and decrease are more prominent in Figure 5b. However, in Figure 5a, the increase of $c_{1}$ is more than the decrease of $c_{2}$, which is different from other results.

## 4. Discussion

### 4.1. Influence of Adjusting Rate k on System Stability

These results suggest that the quantities of low-carbon and high-carbon travelers can achieve a certain equilibrium proportion when the strategy adjustment rate *k* is less than a certain value. On the one hand, from Figure 2 and Figure 4, the upper limits of *k* value of the equilibrium system increase with the enhancement of low-carbon travel awareness. On the other hand, we also find that the upper limit of *k* value of the equilibrium system is much smaller in cities with developed traffic systems than in cities with undeveloped traffic systems. This means that it is easier for the system to maintain a stable equilibrium in cities with undeveloped traffic systems while the most unstable situation is that cities with developed traffic systems are in L-A of low-carbon travel awareness. These results explain the continuous traffic congestion in small and medium-sized cities and the fluctuation of the public transportation share ratio in big cities from another perspective.

Previous studies mainly focused on the share ratio of travel modes and the influencing factors [25,38]. Meanwhile, we discussed the trends and possibilities to achieve a corresponding ratio by game theory and chaos theory, which can be considered as a supplement to the study of travel mode choice.

### 4.2. Effects of Low-Carbon Awareness in Travel Choice

From Figure 2 and Figure 4, the findings suggest that with the enhancement of low-carbon travel awareness, the quantities of low-carbon travelers increase while the quantities of high-carbon travelers decrease. There are no evident differences in the impact of low-carbon travel awareness on the quantities of low-carbon and high-carbon travelers in different types of cities. It should be noted that the increase in the quantities of low-carbon travelers is slightly less than the decrease in the quantities of high-carbon travelers. In other words, high awareness of low-carbon might inhibit traffic demand. Fortunately, the inhibition was negligible in the results.

The results appear to be similar to those reported earlier by some studies [39]. In addition, this paper also points out that the role of low-carbon awareness in cities has a dual character. The quantification of low-carbon awareness and its specific impact on travel need to be further studied.

### 4.3. Measures to Achieve Urban Low-Carbon Traffic

These results agree with some analysis on low-carbon traffic [2,40], in that low-carbon awareness will have impacts on travel choice. However, it is obvious that various types of cities have different low-carbon travel characteristics, and that their ways to achieve low-carbon travel should be different.

From the above analysis, in cities with developed traffic systems, it is difficult to achieve a stable situation of low-carbon travel. Although the enhancement of low-carbon awareness has a significant effect on reducing high-carbon travel, it is still difficult to enhance the stability of the system. Therefore, the focus of low-carbon travel in these cities should be to steadily improve the low-carbon awareness of travelers, and it is not appropriate to make too rapid changes in transportation facilities and policies. On the other hand, in cities with undeveloped traffic systems, it is easier to achieve a stable situation of low-carbon travel and the stable system makes more low-carbon travel options possible. As shown in Figure 5, it is obvious that the effect of decreasing the traditional comprehensive travel costs of low-carbon travel is less than that of increasing the comprehensive costs of high-carbon travel. In addition, it is worth noting that the quantities of high-carbon trips decrease rapidly while the number of low-carbon trips increases slowly, which might lead to the inhibition of some travel willingness. Therefore, small and medium-sized cities with undeveloped traffic systems should be cautious about the construction of high-cost public transport facilities, such as light rail. Although it is beneficial to increase the proportion of low-carbon travel and meet the social traffic demand, these benefits may be relatively limited. However, it is easier for these cities to reach equilibrium of the travel system, and so low-carbon measures are less restricted. These cities should be the most efficient in restricting high-carbon travel to achieve low-carbon travel.

Our research focuses on the macro trend of low-carbon travel choice, which can provide a theoretical basis for decision-making in urban transport development. In the future, we expect to study the traffic game problem from the micro level and get results that are more extensive.

## 5. Conclusions

Grasping the possible states and trend of urban low-carbon traffic development is of great significance for formulating urban transportation policies and reducing traffic carbon emissions. In our study, we collected and collated the data of low-carbon awareness of urban travelers through a questionnaire survey. Then, we built a game model considering low-carbon awareness and analyzed its system stability. Through the analysis of urban traffic conditions, the parameters of the model are calibrated. On this basis, there are some findings through chaos analysis and simulation.

Firstly, cities with undeveloped traffic systems are able to achieve the stability of travel mode choice in any situation of low-carbon awareness. However, it is difficult for cities with developed traffic systems to maintain such stability in L-A of low-carbon awareness. Secondly, in all types of cities, low-carbon awareness will affect the quantity of travelers in different travel modes and there is no significant difference. Finally, according to the different types of cities, we put forward corresponding ways to realize urban low-carbon traffic. In cities with undeveloped traffic systems, the main recommended measure is to increase the comprehensive costs of high-carbon travel. In cities with developed traffic systems, the most efficient way is to raise the low-carbon awareness of travelers. It shows advantages in both adding the quantity of low-carbon travelers and enhancing the stability of travel mode choice. These results provide suggestions for the formulation and adjustment of low-carbon traffic strategies and policies, which can reduce the adverse impact of urban traffic emissions on public health.

## Figures and Tables

**Table 1:** Low-carbon travel awareness of urban travelers.

	Lv 0	Lv 1	Lv 2	Lv 3	Lv 4	Lv 5	Lv 6	Lv 7	Lv 8	Lv 9	Lv 10	Total
Chengdu	33	11	8	11	7	35	9	3	2	1	6	126
Beijing	26	11	6	14	3	41	7	3	7	3	8	129
Nanjing	35	12	15	5	8	35	5	6	5	4	12	142
Zhenjiang	48	19	9	16	13	53	10	3	13	5	16	205
Others	89	38	24	34	14	125	28	11	14	2	30	409
Total	231	91	62	80	45	289	59	26	41	15	72	1011

**Table 2:** Distribution of *w* among urban travelers in various situations.

	Lv 0	Lv 1	Lv 2	Lv 3	Lv 4	Lv 5	Lv 6	Lv 7	Lv 8	Lv 9	Lv10
L-A	29.0%	10.0%	7.0%	8.0%	5.0%	28.0%	3.0%	2.0%	3.0%	1.0%	4.0%
M-A	22.8%	9.0%	6.1%	7.9%	4.5%	28.6%	5.8%	2.6%	4.1%	1.5%	7.1%
H-A	16.0%	6.0%	4.0%	6.0%	3.0%	28.0%	9.0%	6.0%	8.0%	4.0%	10.0%

## Data Availability

Data supporting reported questionnaire survey can be found at https://www.wjx.cn/report/84967665.aspx, accessed on 20 February 2021.
